# Psychotropic Medication Use in Children and Adolescents With Type 1 Diabetes

**DOI:** 10.1001/jamanetworkopen.2023.36621

**Published:** 2023-10-03

**Authors:** Shengxin Liu, Tyra Lagerberg, Jonas F. Ludvigsson, Mark J. Taylor, Zheng Chang, Brian M. D’Onofrio, Henrik Larsson, Paul Lichtenstein, Soffia Gudbjörnsdottir, Ralf Kuja-Halkola, Agnieszka Butwicka

**Affiliations:** 1 Department of Medical Epidemiology and Biostatistics, Karolinska Institutet, Solna, Sweden; 2 Department of Psychiatry, Warneford Hospital, University of Oxford, Oxford, United Kingdom; 3 Department of Paediatrics, Örebro University Hospital, Örebro, Sweden; 4 Department of Medicine, Columbia University College of Physicians and Surgeons, New York, New York; 5 Department of Psychological and Brain Sciences, Indiana University, Bloomington; 6 School of Medical Sciences, Örebro University, Örebro, Sweden; 7 Swedish National Diabetes Register, Centre of Registers, Gothenburg, Sweden; 8 Department of Molecular and Clinical Medicine, Sahlgrenska Academy, University of Gothenburg, Gothenburg, Sweden; 9 Institute of Clinical Medicine, Faculty of Medicine, University of Oslo, Oslo, Norway; 10 Division of Mental Health Services, R&D Department, Akershus University Hospital, Lørenskog, Norway; 11 Department of Biostatistics and Translational Medicine, Medical University of Lodz, Lodz, Poland

## Abstract

**Importance:**

Children and adolescents with type 1 diabetes (T1D) face elevated risks of psychiatric disorders. Despite their nonnegligible adverse effects, psychotropic medications are a common cost-effective approach to alleviating psychiatric symptoms, but evidence regarding their dispensation to children and adolescents with T1D remains lacking.

**Objective:**

To examine the trends and patterns of psychotropic medication dispensation among children and adolescents with T1D in Sweden between 2006 and 2019.

**Design, Setting, and Participants:**

This cohort study used data from multiple Swedish registers. The main study cohort included children and adolescents residing in Sweden from 2006 to 2019 and was followed up until the earliest of December 31, 2019, 18th birthday, emigration, or death. Data analyses were conducted from November 1, 2022, to April 30, 2023.

**Exposures:**

Type 1 diabetes.

**Main Outcomes and Measures:**

The primary outcomes were trends and patterns of psychotropic medication dispensation (including antipsychotics, antidepressants, anxiolytics, hypnotics, mood stabilizers, and medications for attention-deficit/hyperactivity disorder [ADHD]), psychotropic medication initiation, and history of neurodevelopmental and psychiatric diagnosis. Cumulative incidence curves and Cox proportional hazard models were used to estimate the aggregated incidence and hazard ratios of medication initiation after diabetes onset.

**Results:**

Of 3 723 745 children and adolescents (1 896 199 boys [50.9%]), 13 200 (0.4%; 7242 boys [54.9%]) had T1D (median [IQR] age at diagnosis, 11.1 [7.6-14.7] years). Between 2006 and 2019, psychotropic medication dispensation increased from 0.85% (95% CI, 0.65%-1.10%) to 3.84% (3.11%-4.69%) among children and from 2.72% (95% CI, 2.15%-3.39%) to 13.54% (95% CI, 12.88%-14.23%) among adolescents with T1D, consistently higher than their peers without T1D. The most commonly dispensed medications included hypnotics, ADHD medications, anxiolytics, and selective serotonin reuptake inhibitors, and all exhibited increasing trends. For those with T1D, psychiatric care was the primary prescription source, and up to 50.1% of treatments lasted more than 12 months. In addition, children and adolescents with T1D showed higher cumulative incidence and hazard ratios of medication initiation after diabetes onset than their same-age and same-sex counterparts.

**Conclusions and Relevance:**

This cohort study found an increasing trend in psychotropic medication dispensation among children and adolescents with T1D from 2006 to 2019, persistently higher than those without T1D. These findings call for further in-depth investigations into the benefits and risks of psychotropic medications within this population and highlight the importance of integrating pediatric diabetes care and mental health care for early detection of psychological needs and careful monitoring of medication use.

## Key Points

**Question:**

What are the trends and patterns of psychotropic medications used for children and adolescents with type 1 diabetes (T1D)?

**Findings:**

This population-based cohort study of more than 3.7 million Swedish children and adolescents found that, from 2006 to 2019, the dispensation of psychotropic medications, particularly hypnotics, attention-deficit/hyperactivity disorder medication, anxiolytics, and antidepressants, increased from 0.85% to 3.84% among children and from 2.72% to 13.54% among adolescents with T1D. For those with T1D, psychiatric care was the primary prescription source; up to 50.1% of treatments lasted more than 12 months.

**Meaning:**

These findings call for further research to evaluate the benefits and risks of psychotropic medications for children and adolescents with T1D and highlight the need for clinical attentiveness and recommendations.

## Teaser

This cohort study examines the trends and patterns of psychotropic medication dispensation among Swedish children and adolescents with type 1 diabetes between 2006 and 2019.

## Introduction

Type 1 diabetes (T1D) is one of the most common chronic condition onsets during childhood. Children and adolescents with T1D are at elevated risk of psychiatric disorders.^1,2,3,4,5^ Most, if not all, psychiatric disorders pose challenges to diabetes management and adversely affect life-course outcomes.^6,7^

Psychotropic medications are cost-effective and widely used for alleviating psychiatric and neurodevelopmental conditions,^8^ such as depression, anxiety, and attention-deficit/hyperactivity disorder (ADHD). Despite evidence for the efficacy of many psychotropic medications, their potential adverse effects on children and adolescents raise serious concerns. Various antipsychotics, mood stabilizers, and antidepressants have been associated with detrimental metabolic outcomes, including weight gain, hyperlipidemia, and insulin resistance.^9^ Moreover, gastrointestinal issues, such as nausea and appetite changes, are commonly reported adverse effects of antidepressants and ADHD medications.^9^ Furthermore, prolonged use of anxiolytics and hypnotics, particularly benzodiazepines, can also increase the risks of dependence and withdrawal complications.^10^ For children and adolescents with T1D, who already face an increased risk of metabolic complications^11^ and gastrointestinal symptoms,^12^ these adverse effects may exacerbate their health outcomes. Therefore, there is a need for clinical recommendations and/or guidelines for administering psychotropic medications in this population.

To facilitate developing tailored treatment recommendations and directing future research on their risk and benefits, a comprehensive overview of psychotropic medication use among children and adolescents with T1D is crucial. However, few studies provided limited information on this topic. One cohort study^13^ found that approximately 10% of patients with T1D younger than 19 years used psycholeptics and/or psychoanaleptics between 1999 and 2009. Another survey study^14^ reported that 0.48% of patients with T1D younger than 25 years used antipsychotics between 1995 and 2013. Despite these findings, there is a glaring lack of up-to-date, nationally representative research on this topic. Therefore, we conducted this population-based cohort study, using data from Swedish nationwide registers, to explore the trends and patterns, including prescribing sources and treatment durations, of psychotropic medication used in children and adolescents with T1D between 2006 and 2019.

## Methods

### Data Source

For this population-based cohort study, we used data from multiple Swedish registers with nationwide coverage. The Total Population Register was used to identify the study cohort and obtain information on migration and vital status.^15^ Diagnoses of T1D and psychiatric disorders were obtained from the National Patient Register (NPR), using corresponding *International Classification of Diseases, Ninth Revision (ICD-9)* and *International Statistical Classification of Diseases and Related Health Problems, Tenth Revision (ICD-10)* codes.^16^ Information on medication dispensations was acquired from the Prescribed Drug Register (PDR), which documents filled prescriptions from July 1, 2005, using Anatomical Therapeutic Chemical codes.^17^ Details of the registers used are presented in eTable 1 in Supplement 1.

### Study Population

The study cohort included all children (aged 0-11 years) and adolescents (aged 12-17 years) who resided in Sweden at some point between January 1, 2006, and December 31, 2019. We excluded individuals with a diagnosis of congenital malformations (*ICD-9* and *ICD-10* codes are shown in eTable 2 in Supplement 1). We followed up each individual from birth up to their 18th birthday, emigration, death, or end of study (December 31, 2019), whichever came first.

Within the study cohort, we created a matched subsample for estimating the cumulative incidence. For each individual who received a diagnosis of T1D after July 1, 2006, we used an exact matching approach to randomly select 10 individuals, matching on sex and birth year, from those without T1D during the entire follow-up period.

The study followed the Strengthening the Reporting of Observational Studies in Epidemiology (STROBE) reporting guideline. Ethical approval was granted by the Swedish Ethical Review Authority. No informed consent from participants was required for this register-based study because the data were anonymous, in accordance with Swedish regulations.^18^

### Type 1 Diabetes

T1D was identified from the NPR using *ICD-9* code 250 from 1987 to 1996 and* ICD-10* code E10 from 1997 to 2019. The *ICD-9* code (250) used before 1997 does not differentiate between T1D and type 2 diabetes, but type 2 diabetes onset before age 18 years in the Swedish population was infrequent.^19,20^ The implementation of the *ICD-10* code (E10) in 1997 allowed for the explicit identification of T1D afterward.

### Psychotropic Medications

Information for psychotropic medication was obtained from PDR. Examined psychotropic medication included the following groups: first-generation antipsychotics (FGAs), second-generation antipsychotics (SGAs), antidepressants (selective serotonin reuptake inhibitors [SSRIs] and others), anxiolytics, hypnotics, mood stabilizers, and ADHD medication. Detailed types of psychotropic medications included in each group and Anatomical Therapeutic Chemical codes are presented in eTable 3 in Supplement 1.

Consistent with previous studies,^21,22^ when the medication was dispensed continuously, unless 2 dispensations occurred more than 6 months apart, we regarded those dispensations to belong to 1 course of treatment. For each treatment, we obtained information on its prescription source, including primary, nonpsychiatric, and psychiatric specialist care. We calculated the treatment duration and categorized it as short term (<6 months), medium term (6-12 months), and long term (>12 months).

Information on psychiatric and neurodevelopmental disorders diagnosed before or during the year of the treatment was obtained from NPR using *ICD-9* and *ICD-10* codes (eTable 2 in Supplement 1). Diagnoses included depression, bipolar disorder, anxiety disorders, stress-related disorders, psychotic disorders, personality disorders, ADHD, autism spectrum disorder, conduct disorder, substance use disorder, eating disorders, sleep disorders, and epilepsy. When a dispensation occurred after at least 365 days without dispensing that psychotropic medication, we regarded that dispensation as a medication initiation.^23^

### Statistical Analysis

We first reported 14-year (from 2006 to 2019) dispensing trends of any and specific groups of psychotropic medications as annual period prevalence.^24^ The prevalence was calculated for each year as the number of individuals who were dispensed psychotropic medication within a calendar year divided by the total number of individuals who resided in Sweden that year, separately for children and adolescents with and without T1D. We used the Mann-Kendall test to evaluate the presence of temporal changes in the dispensing patterns over the study period.

Among users with T1D, we summarized treatment patterns per psychotropic medication group. The patterns included prescription source, treatment duration, and diagnoses of psychiatric and neurodevelopmental disorders.

In the matched subsample, we plotted cumulative incidence curves to demonstrate the aggregated incidence of initiation of any and specific psychotropic medications from diabetes diagnosis. The start date of the follow-up time was the date of diagnosis for the individual with T1D and his or her matched counterparts. The end date was the date of first recorded medication initiation, 18th birthday, emigration, death, or end of the study, whichever occurred first. We also used stratified Cox proportional hazards models to estimate the hazard ratios (HRs) and 95% CIs for medication initiation, comparing children and adolescents with T1D with their same-age and same-sex peers without T1D. Follow-up time was calculated as described already.

Data management was performed using SAS statistical software version 9.4 (SAS Institute), and analyses were conducted using the survival package in R statistical software version 4.1.2 (R Project for Statistical Computing). Statistical significance was set at 2-tailed *P* < .05. Data analyses were conducted from November 1, 2022, to April 30, 2023.

## Results

We identified 3 723 745 children and adolescents (1 896 199 boys [50.9%]) residing in Sweden during 2006 to 2019, of whom 13 200 (0.4%; 7242 boys [54.9%]) had T1D (median [IQR] age at diagnosis, 11.1 [7.6-14.7] years). Among children and adolescents with T1D, 1866 (14.1%) were dispensed psychotropic medications during 2006 to 2019 (Table 1).

**Table 1.** Characteristic of the Study Cohort

Characteristics	Patients, No. (%)	
	Without type 1 diabetes (n = 3 710 545)	With type 1 diabetes (n = 13 200)
Sex		
Male	1 888 957 (50.9)	7242 (54.9)
Female	1 821 588 (49.1)	5958 (45.1)
Birth cohort		
1989-1994	894 568 (24.1)	2828 (21.4)
1995-2000	676 155 (18.2)	3508 (26.6)
2001-2006	645 332 (17.4)	3906 (29.6)
2007-2012	704 809 (19.0)	2320 (17.6)
2013-2019	789 681 (21.3)	638 (4.8)
Psychotropic medications dispensed during follow-up		
Any psychotropic medication	282 987 (7.6)	1866 (14.1)
First-generation antipsychotics	1683 (<0.1)	17 (0.1)
Second-generation antipsychotics	18 187 (0.5)	102 (0.8)
Selective serotonin reuptake inhibitors	78 852 (2.1)	618 (4.7)
Other antidepressants	16 258 (0.4)	119 (0.9)
Anxiolytics	131 097 (3.5)	755 (5.7)
Hypnotics	115 776 (3.1)	852 (6.5)
Mood stabilizers	22 068 (0.6)	195 (1.5)
Attention-deficit/hyperactivity disorder medication	102 926 (2.8)	735 (5.6)

**Figure 1.**
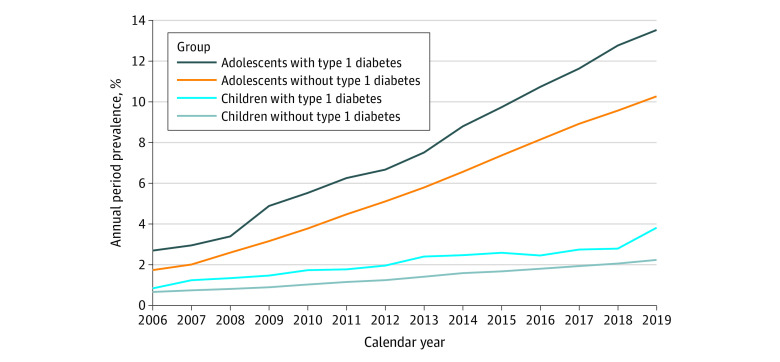
Trends of Annual Period Prevalence of Dispensing Any Psychotropic Medications for Children and Adolescents With and Without Type 1 Diabetes Annual period prevalence (percentage) was calculated as the number of individuals who were dispensed psychotropic medication divided by the total number of individuals who resided in Sweden within 1 calendar year.

The annual period prevalence of dispensing any psychotropic medications increased over the study period (Figure 1). Among children with T1D, the prevalence increased from 0.85% (95% CI, 0.65%-1.10%) in 2006 to 3.84% (95% CI, 3.11%-4.69%) in 2019 (*P *for trend < .001). Among adolescents with T1D, the prevalence increased from 2.72% (95% CI, 2.15%-3.39%) to 13.54% (95% CI, 12.88%-14.23%) (*P *for trend < .001). The prevalence was consistently higher among those with T1D than among those without, for whom the prevalence increased from 0.66% (95% CI, 0.65%-0.68%) to 2.29% (95% CI, 2.27%-2.32%) among children and from 1.77% (95% CI, 1.74%-1.79%) to 10.29% (95% CI, 10.28%-10.36%) among adolescents (both *P *for trend < .001).

**Figure 2.**
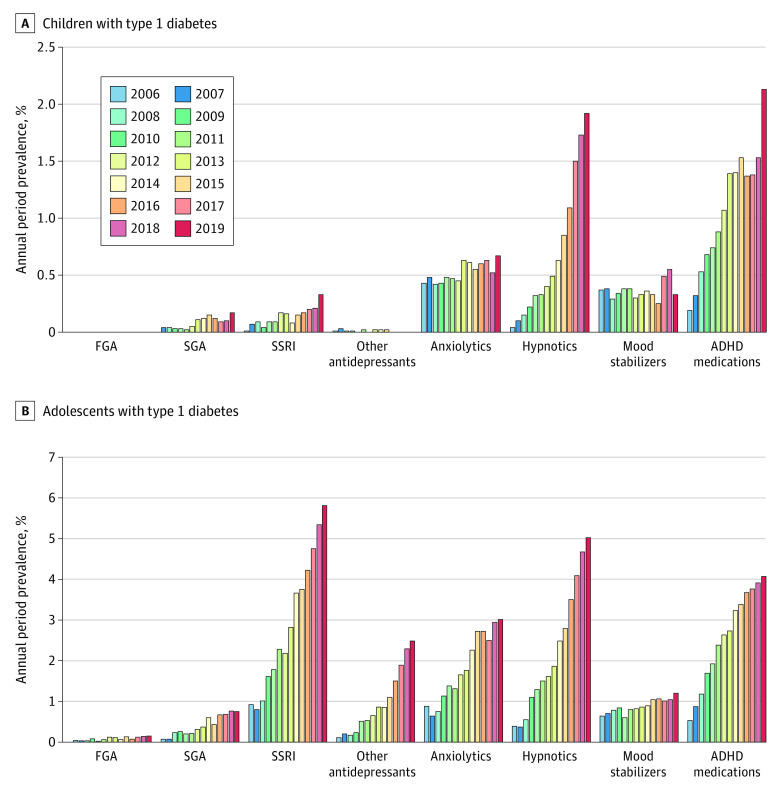
Annual Period Prevalence of Dispensing Specific Group of Psychotropic Medications, 2006-2019 Annual period prevalence (percentage) was calculated as the number of individuals who were dispensed psychotropic medication divided by the total number of individuals who resided in Sweden within 1 calendar year. Graphs show data for children (A) and adolescents (B) with type 1 diabetes. ADHD indicates attention-deficit/hyperactivity disorder; FGA, first-generation antipsychotic; SGA, second-generation antipsychotic; and SSRI, selective serotonin reuptake inhibitor.

Regarding specific medication groups (Figure 2), among children with T1D, increases in annual period prevalence were observed for SSRI (from 0.01% to 0.33%; *P* for trend < .001), hypnotics (0.04% to 1.92%; *P* for trend < .001), ADHD medication (from 0.19% to 2.13%; *P* for trend, <.001), and anxiolytics (from 0.43% to 0.67%; *P* for trend = .01). Dispensation of mood stabilizers remained stably high in children with T1D, with a mean prevalence of 0.36%. The prevalence of SGA remained low, despite its estimate increase from 0.00% to 0.17%, and there was almost no dispensation of FGA and other antidepressants. Among adolescents with T1D, notably increased prevalence was observed for all medication groups, including FGA (from 0.04% to 0.15%; *P* for trend = .003), SGA (from 0.07% to 0.75%; *P* for trend < .001), SSRI (from 0.92% to 5.81%; *P* for trend < .001), other antidepressants (from 0.11% to 2.48%; *P* for trend < .001), hypnotics (from 0.39% to 5.02%; *P* for trend < .001), anxiolytics (from 0.88% to 3.01%; *P* for trend < .001), mood stabilizers (from 0.64% to 1.20%; *P* for trend < .001), and ADHD medication (from 0.53% to 4.07%; *P* for trend < .001). These prevalences were constantly higher than those for children and adolescents without T1D (eTable 4 in Supplement 1). Notably, by the year 2019, the prevalence of ADHD medication dispensation was comparable in adolescents with and without T1D (5.85% vs 4.37%) but was much higher in children with T1D than those without T1D (2.07% vs 0.91%).

For psychotropic medications dispensed for children and adolescents with T1D, the most common prescription source was psychiatric care for antipsychotics (138 of 186 prescriptions [74.2%]), antidepressants (888 of 1122 prescriptions [79.1%]), hypnotics (911 of 1597 prescriptions [57.0%]), and ADHD medication (977 of 1374 prescriptions [71.1%]), and nonpsychiatric specialist for mood stabilizers (570 of 618 prescriptions [92.2%]) (Table 2); anxiolytics were prescribed comparably frequently from all 3 sources (primary care, psychiatric care, and nonpsychiatric specialist). Most psychotropic medication treatments were short term (<6 months) and medium term (6-12 months), with several exceptions. FGAs (13 of 21 prescriptions [61.9%]) and anxiolytics (581 of 1037 prescriptions [56.0%]) were typically dispensed as a single dispensation, and dispensations of ADHD medication (688 of 1374 prescriptions [50.1%]) mainly were long term (>12 months). Notably, a substantial proportion of SGAs (54 of 165 prescriptions [32.7%]), SSRIs (299 of 975 prescriptions [30.7%]), hypnotics (489 of 1597 prescriptions [30.6%]), and anxiolytics (207 of 1037 prescriptions [20.0%]) were also prescribed long term. Unipolar depression, anxiety disorders, and ADHD were the most common diagnoses among users with T1D, except that epilepsy was the most common diagnosis (138 of 195 patients [70.8%]) for users of mood stabilizers. Detailed numbers of distinct dispensations of each type of psychotropic medication are presented in eTable 5 in Supplement 1. Notably, 80.1% of prescribed mood stabilizers were valporate and lamotrigine, and melatonin constituted 84.1% of the prescribed hypnotics.

**Table 2.** Patterns of Psychotropic Medications and Diagnoses of Psychiatric and Neurodevelopmental Disorders in Users With Type 1 Diabetes, 2006-2019

Variable	Patients, No. (%)							
	FGA	SGA	SSRI	Other antidepressants	Anxiolytics	Hypnotics	Mood stabilizers	ADHD medication
Total No. of distinct prescriptions	21	165	975	147	1037	1597	618	1374
Total No. of users	17	102	618	119	755	852	195	735
Prescription sources								
Primary care only	<5^a^	7 (4.2)	55 (5.6)	17 (11.6)	213 (20.5)	81 (5.1)	11 (1.8)	13 (0.9)
Psychiatric specialist care	13 (61.9)	125 (75.8)	794 (81.4)	94 (63.9)	361 (34.8)	911 (57.0)	33 (5.3)	977 (71.1)
Nonpsychiatric care	5 (23.8)	28 (17.0)	87 (8.9)	33 (22.4)	451 (43.5)	535 (33.5)	570 (92.2)	306 (22.3)
All sources	0	5 (3.0)	39 (4.0)	<5^a^	12 (1.2)	70 (4.4)	<5^a^	78 (5.7)
Treatment duration								
Single prescription^b^	13 (61.9)	28 (17.0)	138 (14.2)	53 (36.1)	581 (56.0)	431 (27.0)	60 (9.7)	59 (4.3)
Short term (<6 mo)	<5^a^	38 (23.0)	247 (25.3)	44 (29.9)	126 (12.2)	260 (16.3)	75 (12.1)	224 (16.3)
Medium term (6-12 mo)	<5^a^	45 (27.3)	291 (29.8)	25 (17.0)	123 (11.9)	417 (26.1)	252 (40.8)	403 (29.3)
Long term (>12 mo)	<5^a^	54 (32.7)	299 (30.7)	25 (17.0)	207 (20.0)	489 (30.6)	231 (37.4)	688 (50.1)
Psychiatric and neurodevelopmental disorder diagnoses^c^								
Unipolar depression	<5^a^	24 (23.5)	325 (52.6)	58 (48.7)	156 (20.7)	243 (28.5)	21 (10.8)	109 (14.8)
Anxiety disorders	<5^a^	43 (42.2)	296 (47.9)	47 (39.5)	186 (24.6)	220 (25.8)	21 (10.8)	103 (14.0)
Stress-related disorders	<5^a^	12 (11.8)	73 (11.8)	15 (12.6)	58 (7.7)	76 (8.9)	12 (6.2)	45 (6.1)
ADHD	7 (41.2)	45 (44.1)	170 (27.5)	34 (28.6)	125 (16.6)	339 (39.8)	42 (21.5)	643 (87.5)
Autism spectrum disorder	<5^a^	31 (30.4)	97 (15.7)	14 (11.8)	63 (8.3)	157 (18.4)	26 (13.3)	136 (18.5)
Disruptive behavior disorder	<5^a^	14 (13.7)	27 (4.4)	<5^a^	25 (3.3)	48 (5.6)	10 (5.1)	57 (7.8)
Sleep disorders	<5^a^	8 (7.8)	51 (8.3)	11 (9.2)	45 (6.0)	107 (12.6)	8 (4.1)	43 (5.9)
Eating disorder	<5^a^	12 (11.8)	52 (8.4)	10 (8.4)	31 (4.1)	36 (4.2)	<5^a^	12 (1.6)
Substance use disorder	<5^a^	14 (13.7)	47 (7.6)	18 (15.1)	40 (5.3)	57 (6.7)	9 (4.6)	42 (5.7)
Bipolar disorder	0	<5^a^	<5^a^	<5^a^	<5^a^	5 (0.6)	7 (3.6)	5 (0.7)
Personality disorder	<5^a^	7 (6.9)	21 (3.4)	8 (6.7)	15 (2.0)	22 (2.6)	<5^a^	13 (1.8)
Epilepsy	<5^a^	<5^a^	12 (1.9)	0	89 (11.8)	79 (9.3)	138 (70.8)	23 (3.1)

Abbreviations: ADHD, attention-deficit/hyperactivity disorder; FGA, first-generation antipsychotic; SGA, second-generation antipsychotic; SSRI, selective serotonin reuptake inhibitor.

^a^ In accordance with Swedish register guidelines, exact numbers are not reported for cells with fewer than 5 patients.

^b^ Single prescription entails single dispensed prescriptions for that medication group. This number does not capture switching between different types of medications within that group.

^c^ Refers to psychiatric and neurological diagnoses made during or before the year of the start of that distinct treatment.

**Figure 3.**
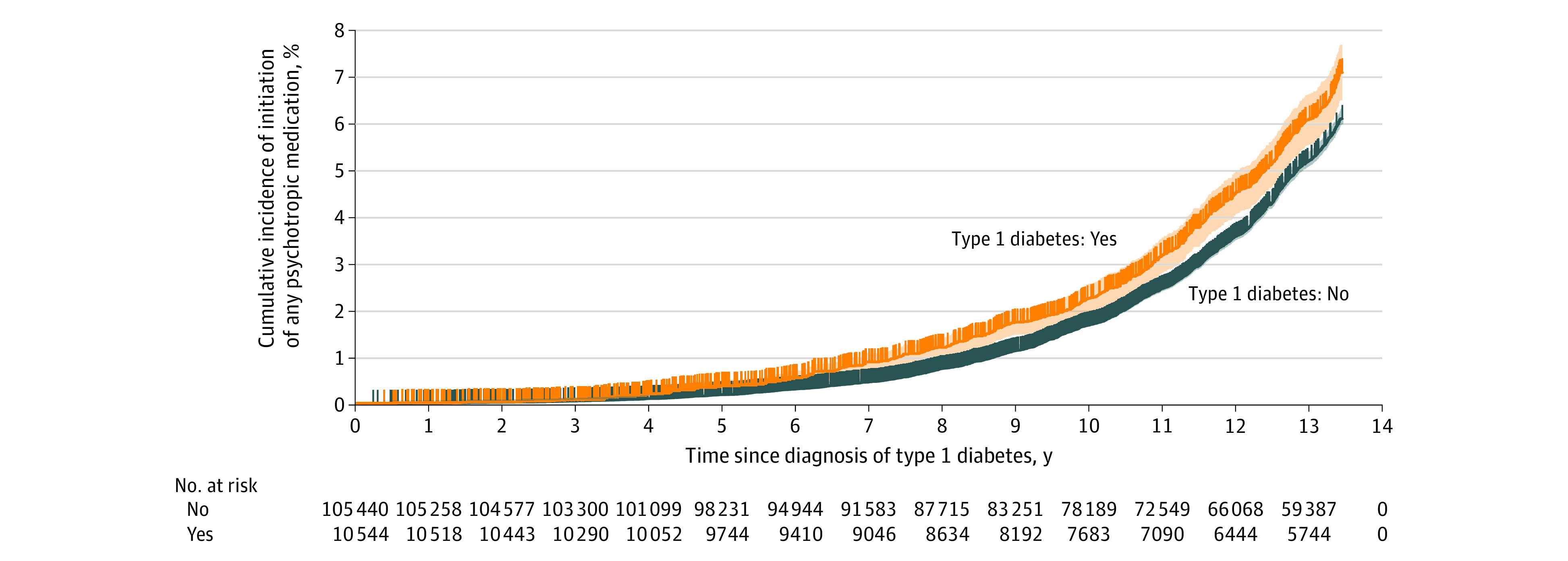
Survival Curve of Initiation of Psychotropic Medications for Children and Adolescents With Childhood-Onset Type 1 Diabetes Compared With Sex-Matched and Age-Matched Reference Individuals Without Type 1 Diabetes Psychotropic medication initiation was defined as a dispensation that occurred by at least 365 days without the dispensation of that medication.

Figure 3 shows the cumulative incidences of any medication initiation among children and adolescents with T1D diagnosed after July 1, 2006, compared with their sex-matched and age-matched counterparts. The difference in proportion started to be visible approximately 3 years after diabetes diagnosis and increased steadily. Approximately 13 years after diabetes diagnosis, accounting for censoring, 6.8% (95% CI, 6.2%-7.3%) of individuals with T1D had initiated psychotropic medication, compared with 5.7% (95% CI, 5.6%-5.9%) of the matched individuals. The gradually increased proportion differences were also observed for SSRIs, anxiolytics, hypnotics, mood stabilizers, and ADHD medications (eFigure in Supplement 1).

Results from Cox models supported these findings, where those with T1D had a 1.21-fold increased risk of initiating any psychotropic medication (HR, 1.21; 95% CI, 1.11-1.32) compared with the sex-matched and age-matched reference group. Statistically significantly elevated risks were also observed for SSRIs (HR, 1.50; 95% CI, 1.29-1.73), other antidepressants (HR, 1.70; 95% CI, 1.25-2.31), anxiolytics (HR, 1.22; 95% CI, 1.07-1.40), hypnotics (HR, 1.43; 95% CI, 1.28-1.59), mood stabilizers (HR, 2.85; 95% CI, 2.06-3.96), and ADHD medication (HR, 1.21; 95% CI, 1.06-1.39) (eTable 6 in Supplement 1).

## Discussion

To our knowledge, this is the largest nationwide population-based cohort study examining psychotropic medication use among children and adolescents with T1D. From 2006 to 2019, we observed a noteworthy and steady increase in psychotropic medication dispensations, with the annual period prevalence of any and specific psychotropic medication consistently higher among those with T1D than those without T1D over the follow-up period. Children and adolescents with T1D were more likely to initiate psychotropic medications, specifically SSRI, anxiolytics, hypnotics, mood stabilizers, and ADHD medication, than their sex-matched and age-matched counterparts.

Our observed trends in the annual period prevalence of psychotropic medications dispensed for children and adolescents with T1D resemble the trends observed in the general pediatric populations across countries.^25,26,27^ Only 1 Dutch study^13^ explicitly reported psychotropic medication use among pediatric patients with T1D. The study found that 10.4% of individuals, who had at least 2 insulin dispensations before age 19 years, had been dispensed psycholeptics and/or psychoanaleptics during 1999 to 2009. This estimate is lower than the 14.1% estimated in our study sample, which might be explained by the changes in clinical practices in the recent decade (eg, updates in approved drugs and guidelines for psychotropic medication use in the pediatric population).

Concerning antipsychotics, we observed similar trends—a relatively steady low prevalence of FGA and an increased prevalence of SGA, particularly among adolescents—as in recent studies from European countries.^24,28^ Notably, the prevalence of antipsychotics was slightly higher in those with than those without T1D. A previous study^14^ using data from the German/Austrian Diabetes Survey showed that patients with T1D (aged <25 years) using antipsychotics, especially SGA, had worse glycemic control and more diabetic complications than the nonusers. Although antipsychotics sometimes can be necessary for managing severe mental illness and are increasingly used in managing challenging behaviors in pediatric patients with ADHD or ASD,^29^ it is worth repeating that, for children and adolescents with T1D, prudent prescribing and close monitoring considering the metabolic effects are needed.

SSRIs, anxiolytics, and hypnotics were among the most prevalently used psychotropic medications in our study sample, with increased trends throughout the follow-up. This observation is consistent with the high prevalence of symptoms of depression, anxiety, and sleep problems found in children and adolescents with T1D.^30,31^ Most of these medications were dispensed as a single dispensation or continued treatment for less than 12 months, which reflects recommendations from current pediatric psychiatric guidelines^8^; however, 30.7% of SSRIs, 30.6% of hypnotics, and 20.0% of anxiolytics were dispensed regularly over an extended period of more than 12 months. This finding raises questions on whether these patients experienced severe and persistent symptoms that required intensive treatment and whether such long-term treatments can interfere with their diabetes management. In addition, melatonin constituted 84.1% of the prescribed hypnotics, most of which were used with long-term duration. Although melatonin is considered relatively safe, there remains a lack of information on its long-term safety in children and adolescents, let alone in those with T1D.

Of note, there was a nearly 10-fold increase in ADHD medication dispensation in children and adolescents with T1D from 2006 to 2019. By the year 2019, the prevalence was comparable in adolescents with and without T1D (5.85% vs 4.37%) but was much higher in children with T1D than those without (2.07% vs 0.91%). This finding agreed with previous findings on the elevated risk of ADHD in T1D,^32^ but this may be explained by the diagnostic bias, where those with T1D were more likely to receive a timely diagnosis because of their frequent contact with health care practitioners.

Although the proportion of mood stabilizers was relatively high at the start of follow-up (2006) compared with other medications, the proportion remained largely unchanged over the years. When taking a closer look, we observed that most mood stabilizers were prescribed in nonpsychiatric specialized care (92.2%) (eg, pediatric care), with valproate and lamotrigine being the most prescribed ones (eTable 5 in Supplement 1), and 70.8% of the users had epilepsy. This finding suggests that the mood stabilizers dispensed by our study sample are more likely to treat epilepsy and further reflect the increased comorbidity between epilepsy and T1D in pediatric patients.^33^

The cumulative incidence curves revealed that a higher percentage of children and adolescents with T1D began taking medications approximately 3 years after diagnosis compared with their peers of the same age and sex who did not have T1D. This difference in proportion also increased over time. Results from Cox models further supported that having T1D was statistically significantly associated with increased risks of medication initiation, after accounting for age and sex. A possible explanation is that, even at a young age, having T1D places the affected children and adolescents at risk of psychiatric conditions that require psychotropic medication. An alternative explanation could be that those with T1D have more frequent medical visits and opportunities for psychological screening and timely treatment.

Psychotropic medication use can be a double-edged sword for children and adolescents with T1D and psychiatric morbidity. On the one hand, psychotropic medications could alleviate symptoms of psychiatric disorders, which may subsequently improve the patient’s diabetes management. For instance, clinical practices showed that antidepressant treatment could improve glycemic control in those with either depressive symptoms or depression.^34^ A recent cross-sectional study^35^ also found that among adults with comorbid T1D and ADHD, those taking ADHD medication had better diabetes outcomes than those with untreated ADHD. On the other hand, the metabolic effects of psychotropic medications, especially antipsychotics and mood stabilizers, have been of concern in deteriorating the metabolic profile of pediatric patients. In addition, treatments with antipsychotics and antidepressants have also been linked to an increased risk of diabetic ketoacidosis and hypoglycemia.^36,37^ In addition, the appetite change and gastrointestinal adverse effects induced by certain antidepressants and ADHD medication^9^ may be challenging for maintaining stable glycemic control. Future longitudinal studies are warranted to assess the effectiveness of psychotropic medication with respect to improving psychiatric symptoms and diabetes management and, at the same time, accounting for potential impacts on metabolic profile. Moreover, the high comorbidity among psychiatric disorders underscores the need for further investigation into the combined effects of multiple disorders and the concurrent usage of different psychotropic medications.

The findings from our study emphasize the importance of integrating pediatric diabetes care and mental health professionals when managing children and adolescents with T1D. This collaborative effort is essential not only for the early detection and screening of patients’ psychological needs but also for the diligent monitoring of psychotropic medication usage and patient outcomes. Given the rapidly growing proportion of children and adolescents with T1D using psychotropic medications, active and close follow-up is required to evaluate treatment effectiveness and carefully monitor their glycemic control and metabolic profiles. In addition, regular reassessments are crucial to determine the most appropriate types and durations of psychotropic medications to ensure the best possible outcomes. Furthermore, developing evidence-based strategies and best practices will ensure that health care practitioners are well-equipped to address the unique challenges faced by the affected children and adolescents, ultimately leading to better health outcomes and improved quality of life.

### Limitations

This study has several limitations that need to be noted. First, we used dispensation records from PDR as proxies for medication use but could not verify whether the dispensed medications were consumed. Second, we did not have information on precise medical indications for dispensations or diagnoses made in primary care. Third, these results were derived from Sweden, which has free-of-charge medical care for all children and adolescents, and the findings may not necessarily be generalized to other countries.

## Conclusions

In this cohort study of children and adolescents residing in Sweden, we observed a noteworthy increase in psychotropic medication use, especially antidepressants, hypnotics, ADHD medications, and anxiolytics, among those with T1D from 2006 to 2019, higher than that for those without T1D. Given the ongoing debate about psychopharmacological treatments in this population, these increases call for careful monitoring and thorough risk-benefit studies to better evaluate medication effectiveness and improve pediatric diabetes care.

## Notes

Supplement 1. **eTable 1.** Details on Used Swedish National Registers **eTable 2.** Details on Used *International Classification of Diagnoses* (*ICD*) Codes **eTable 3.** Details on Used Anatomical Therapeutic Chemical (ATC) Codes **eTable 4.** Annual Period Prevalence of Any and Specific Group of Psychotropic Medication Dispensed Among Children (Age 0-11) and Adolescents With Type 1 Diabetes With and Without Type 1 Diabetes From 2006 to 2019 **eFigure.** Survival Curve of Initiating Specific Psychotropic Medications for Children and Adolescents Diagnosed With Childhood-Onset Type 1 Diabetes Compared With Sex- and Age-Matched Reference Individuals Without Type 1 Diabetes **eTable 5.** Risk of Initiating Psychotropic Medications for Children and Adolescents Diagnosed With Type 1 Diabetes Compared to Sex- and Age-Matched Reference Individuals Without Type 1 Diabetes **eTable 6.** Number of Distinct Dispensations of Each Type of Psychotropic Medications in Children and Adolescents With Type 1 Diabetes
eReferences

Supplement 2.
Data Sharing Statement

## References

[1] Benton M, Cleal B, Prina M, . Prevalence of mental disorders in people living with type 1 diabetes: a systematic literature review and meta-analysis. Gen Hosp Psychiatry. 2023;80:1-16. doi:10.1016/j.genhosppsych.2022.11.004 36493531

[2] de Wit M, Gajewska KA, Goethals ER, . ISPAD Clinical Practice Consensus Guidelines 2022: psychological care of children, adolescents and young adults with diabetes. Pediatr Diabetes. 2022;23(8):1373-1389. doi:10.1111/pedi.13428 36464988 PMC10107478

[3] Dybdal D, Tolstrup JS, Sildorf SM, . Increasing risk of psychiatric morbidity after childhood onset type 1 diabetes: a population-based cohort study. Diabetologia. 2018;61(4):831-838. doi:10.1007/s00125-017-4517-7 29242985

[4] Cooper MN, Lin A, Alvares GA, de Klerk NH, Jones TW, Davis EA. Psychiatric disorders during early adulthood in those with childhood onset type 1 diabetes: rates and clinical risk factors from population-based follow-up. Pediatr Diabetes. 2017;18(7):599-606. doi:10.1111/pedi.12469 27878933

[5] Butwicka A, Frisén L, Almqvist C, Zethelius B, Lichtenstein P. Risks of psychiatric disorders and suicide attempts in children and adolescents with type 1 diabetes: a population-based cohort study. Diabetes Care. 2015;38(3):453-459. doi:10.2337/dc14-0262 25650362 PMC4338504

[6] Ducat L, Rubenstein A, Philipson LH, Anderson BJ. A review of the mental health issues of diabetes conference. Diabetes Care. 2015;38(2):333-338. doi:10.2337/dc14-1383 25614689 PMC4302262

[7] Sildorf SM, Breinegaard N, Lindkvist EB, . Poor metabolic control in children and adolescents with type 1 diabetes and psychiatric comorbidity. Diabetes Care. 2018;41(11):2289-2296. doi:10.2337/dc18-0609 30270201

[8] Smogur M, Onesanu A, Plessen KJ, Eap CB, Ansermot N. Psychotropic drug prescription in children and adolescents: approved medications in European countries and the United States. J Child Adolesc Psychopharmacol. 2022;32(2):80-88. doi:10.1089/cap.2021.0027 35138922

[9] Solmi M, Fornaro M, Ostinelli EG, . Safety of 80 antidepressants, antipsychotics, anti-attention-deficit/hyperactivity medications and mood stabilizers in children and adolescents with psychiatric disorders: a large scale systematic meta-review of 78 adverse effects. World Psychiatry. 2020;19(2):214-232. doi:10.1002/wps.20765 32394557 PMC7215080

[10] Darker CD, Sweeney BP, Barry JM, Farrell MF, Donnelly-Swift E. Psychosocial interventions for benzodiazepine harmful use, abuse or dependence. Cochrane Database Syst Rev. 2015;5:CD009652. doi:10.1002/14651858.CD009652.pub2 26106751 PMC11023022

[11] Kilpatrick ES, Rigby AS, Atkin SL. Insulin resistance, the metabolic syndrome, and complication risk in type 1 diabetes: “double diabetes” in the Diabetes Control and Complications Trial. Diabetes Care. 2007;30(3):707-712. doi:10.2337/dc06-1982 17327345

[12] De Melo EN, Clarke ABM, McDonald C, . Gastrointestinal symptoms in type 1 diabetes: relationship with autoimmune and microvascular complications. J Clin Endocrinol Metab. 2022;107(6):e2431-e2437. doi:10.1210/clinem/dgac093 35176765

[13] Fazeli Farsani S, Abdullah-Koolmees H, Souverein PC, de Boer A, Mantel-Teeuwisse AK. Psychiatric medication use before and after the onset of type 1 diabetes in children and adolescents: a population-based cohort study. Pediatr Diabetes. 2018;19(1):121-128. doi:10.1111/pedi.12529 28436135

[14] Galler A, Bollow E, Meusers M, ; German Federal Ministry of Education and Research (BMBF) Competence Network Diabetes Mellitus. Comparison of glycemic and metabolic control in youth with type 1 diabetes with and without antipsychotic medication: analysis from the nationwide German/Austrian Diabetes Survey (DPV). Diabetes Care. 2015;38(6):1051-1057. doi:10.2337/dc14-2538 25784664

[15] Ludvigsson JF, Almqvist C, Bonamy A-KE, . Registers of the Swedish total population and their use in medical research. Eur J Epidemiol. 2016;31(2):125-136. doi:10.1007/s10654-016-0117-y 26769609

[16] Ludvigsson JF, Andersson E, Ekbom A, . External review and validation of the Swedish national inpatient register. BMC Public Health. 2011;11(1):450. doi:10.1186/1471-2458-11-450 21658213 PMC3142234

[17] Wettermark B, Hammar N, Fored CM, . The new Swedish Prescribed Drug Register—opportunities for pharmacoepidemiological research and experience from the first six months. Pharmacoepidemiol Drug Saf. 2007;16(7):726-735. doi:10.1002/pds.1294 16897791

[18] Ludvigsson JF, Håberg SE, Knudsen GP, . Ethical aspects of registry-based research in the Nordic countries. Clin Epidemiol. 2015;7:491-508. doi:10.2147/CLEP.S90589 26648756 PMC4664438

[19] Mollazadegan K, Kugelberg M, Montgomery SM, Sanders DS, Ludvigsson J, Ludvigsson JF. A population-based study of the risk of diabetic retinopathy in patients with type 1 diabetes and celiac disease. Diabetes Care. 2013;36(2):316-321. doi:10.2337/dc12-0766 22966098 PMC3554314

[20] Nationaella Diabetesregistret. Swediabkids: Swedish National Diabetes Register (NDR), diabetes in children and adolescents—annual report 2020. 2020. Accessed August 30, 2023. https://registercentrum.blob.core.windows.net/refdocs/10.18158/B1gVOc_w-q.pdf

[21] Furu K, Wettermark B, Andersen M, Martikainen JE, Almarsdottir AB, Sørensen HT. The Nordic countries as a cohort for pharmacoepidemiological research. Basic Clin Pharmacol Toxicol. 2010;106(2):86-94. doi:10.1111/j.1742-7843.2009.00494.x 19961477

[22] Lagerberg T, Molero Y, D’Onofrio BM, . Antidepressant prescription patterns and CNS polypharmacy with antidepressants among children, adolescents, and young adults: a population-based study in Sweden. Eur Child Adolesc Psychiatry. 2019;28(8):1137-1145. doi:10.1007/s00787-018-01269-2 30659386 PMC6675912

[23] Lagerberg T, Fazel S, Sjölander A, Hellner C, Lichtenstein P, Chang Z. Selective serotonin reuptake inhibitors and suicidal behaviour: a population-based cohort study. Neuropsychopharmacology. 2022;47(4):817-823. doi:10.1038/s41386-021-01179-z 34561608 PMC8882171

[24] Radojčić MR, Pierce M, Hope H, . Trends in antipsychotic prescribing to children and adolescents in England: cohort study using 2000-19 primary care data. Lancet Psychiatry. 2023;10(2):119-128. doi:10.1016/S2215-0366(22)00404-7 36638816

[25] Piovani D, Clavenna A, Bonati M. Prescription prevalence of psychotropic drugs in children and adolescents: an analysis of international data. Eur J Clin Pharmacol. 2019;75(10):1333-1346. doi:10.1007/s00228-019-02711-3 31270564

[26] Barczyk ZA, Rucklidge JJ, Eggleston M, Mulder RT. Psychotropic medication prescription rates and trends for New Zealand children and adolescents 2008–2016. J Child Adolesc Psychopharmacol. 2020;30(2):87-96. doi:10.1089/cap.2019.0032 31633377

[27] Klau J, Bernardo CO, Gonzalez-Chica DA, Raven M, Jureidini J. Trends in prescription of psychotropic medications to children and adolescents in Australian primary care from 2011 to 2018. Aust N Z J Psychiatry. 2022;56(11):1477-1490. doi:10.1177/00048674211067720 34963342

[28] Kaguelidou F, Holstiege J, Schink T, . Use of antipsychotics in children and adolescents: a picture from the ARITMO population-based European cohort study. Epidemiol Psychiatr Sci. 2020;29:e117. doi:10.1017/S2045796020000293 32308179 PMC7214736

[29] Shafiq S, Pringsheim T. Using antipsychotics for behavioral problems in children. Expert Opin Pharmacother. 2018;19(13):1475-1488. doi:10.1080/14656566.2018.1509069 30102079

[30] Buchberger B, Huppertz H, Krabbe L, Lux B, Mattivi JT, Siafarikas A. Symptoms of depression and anxiety in youth with type 1 diabetes: a systematic review and meta-analysis. Psychoneuroendocrinology. 2016;70:70-84. doi:10.1016/j.psyneuen.2016.04.019 27179232

[31] Caruso NC, Radovanovic B, Kennedy JD, . Sleep, executive functioning and behaviour in children and adolescents with type 1 diabetes. Sleep Med. 2014;15(12):1490-1499. doi:10.1016/j.sleep.2014.08.011 25441750

[32] Liu S, Kuja-Halkola R, Larsson H, . Poor glycaemic control is associated with increased risk of neurodevelopmental disorders in childhood-onset type 1 diabetes: a population-based cohort study. Diabetologia. 2021;64(4):767-777. doi:10.1007/s00125-020-05372-5 33454829 PMC7940269

[33] Fazeli Farsani S, Souverein PC, van der Vorst MM, Knibbe CA, de Boer A, Mantel-Teeuwisse AK. Chronic comorbidities in children with type 1 diabetes: a population-based cohort study. Arch Dis Child. 2015;100(8):763-768. doi:10.1136/archdischild-2014-307654 25877155

[34] Snoek FJ, Bremmer MA, Hermanns N. Constructs of depression and distress in diabetes: time for an appraisal. Lancet Diabetes Endocrinol. 2015;3(6):450-460. doi:10.1016/S2213-8587(15)00135-7 25995123

[35] Vinker-Shuster M, Eldor R, Green I, Golan-Cohen A, Manor I, Merzon E. Glycemic control and diabetes related complications in adults with type 1 diabetes mellitus and ADHD. J Atten Disord. 2022;26(9):1235-1244. doi:10.1177/10870547211068039 34933573

[36] Polcwiartek C, Kragholm K, Rohde C, Hashemi N, Vang T, Nielsen J. Diabetic ketoacidosis and diabetes associated with antipsychotic exposure among a previously diabetes-naive population with schizophrenia: a nationwide nested case-control study. Diabetologia. 2017;60(9):1678-1690. doi:10.1007/s00125-017-4320-5 28593353

[37] Roopan S, Larsen ER. Use of antidepressants in patients with depression and comorbid diabetes mellitus: a systematic review. Acta Neuropsychiatr. 2017;29(3):127-139. doi:10.1017/neu.2016.54 27776567

