# Supplementary material for: Psychotropic Medication Use in Children and Adolescents With Type 1 Diabetes
Source: JAMA Netw Open. 2023 Oct 3;6(10):e2336621. doi: 10.1001/jamanetworkopen.2023.36621 (PMC10548296; doi:10.1001/jamanetworkopen.2023.36621)
Supplement: Supplement 1. — eTable 1. Details on Used Swedish National Registers eTable 2. Details on Used International Classification of Diagnoses (ICD) Codes eTable 3. Details on Used Anatomical Therapeutic Chemical (ATC) Codes eTable 4. Annual Period Prevalence of Any and Specific Group of Psychotropic Medication Dispensed Among Children (Age 0-11) and Adolescents With Type 1 Diabetes With and Without Type 1 Diabetes From 2006 to 2019 eFigure. Survival Curve of Initiating Specific Psychotropic Medications for Children and Adolescents Diagnosed With Childhood-Onset Type 1 Diabetes Compared With Sex- and Age-Matched Reference Individuals Without Type 1 Diabetes eTable 5. Risk of Initiating Psychotropic Medications for Children and Adolescents Diagnosed With Type 1 Diabetes Compared to Sex- and Age-Matched Reference Individuals Without Type 1 Diabetes eTable 6. Number of Distinct Dispensations of Each Type of Psychotropic Medications in Children and Adolescents With Type 1 Diabetes eReferences [file jamanetwopen-e2336621-s001.pdf]

Supplemental Online Content

Liu S, Lagerberg T, Ludvigsson JF, et al. Psychotropic medication use in children and adolescents with type 1 diabetes. *JAMA Netw Open*. 2023;6(10):e2336621. doi:10.1001/jamanetworkopen.2023.36621

eTable 1. Details on Used Swedish National Registers

eTable 2. Details on Used *International Classification of Diagnoses* (*ICD*) Codes

eTable 3. Details on Used Anatomical Therapeutic Chemical (ATC) Codes

eTable 4. Annual Period Prevalence of Any and Specific Group of Psychotropic Medication Dispensed Among Children (Age 0-11) and Adolescents With Type 1 Diabetes With and Without Type 1 Diabetes From 2006 to 2019

eFigure. Survival Curve of Initiating Specific Psychotropic Medications for Children and Adolescents Diagnosed With Childhood-Onset Type 1 Diabetes Compared With Sex- and Age-Matched Reference Individuals Without Type 1 Diabetes

eTable 5. Number of Distinct Dispensations of Each Type of Psychotropic Medications in children and Adolescents With Type 1 Diabetes

eTable 6. Risk of Initiating Psychotropic Medications for children and Adolescents Diagnosed With Type 1 Diabetes Compared to sex- and Age-Matched Reference Individuals Without Type 1 Diabetes

eReferences

This supplemental material has been provided by the authors to give readers additional information about their work.

© 2023 Liu S et al. *JAMA Network Open.*

eTable 1. Details on Used Swedish National Registers

Register name (coverage period)	Summary of available information
Total Population Register (1961-)	The Total Population Register covers the complete Swedish population and contains information on their life events, including birth, death, marital status, and migration [1].
National Patient Register (1964-)	The National Patient Register provides information on inpatient (nationwide coverage since 1987) and outpatient (since 2001) specialist healthcare facilities. Diagnoses were coded using the International Classification of Diseases (ICD 7-10) and were given by a consultant physician at the time of discharge. The validity of many diagnoses has been evaluated and found to be 95-100% [2].
The Prescribed Drug Register (July 2005 -)	The prescribed drug register has complete coverage of prescriptions in Sweden since July 2005, coded according to the Anatomical Therapeutic Chemical (ATC) classification system [3].

© 2023 Liu S et al. *JAMA Network Open.*

eTable 2. Details on Used *International Classification of Diagnoses* (*ICD*) Codes

**Diagnoses**	**ICD-9**<br>**1987-1996**	**ICD-10**<br>**1997-**
**Type 1 diabetes**	250	E10
**Neuropsychiatric disorders**		
Unipolar depression	296B, 300E, 311	F32, F33, F34, F39
Bipolar disorder	296 (excluding 296B)	F30, F31
Anxiety disorders	300A, 300C, 300D, 300X	F40, F41, F42
Stress-related disorders	308-309	F43
Psychosis episodes	295 (excluding 295H)	F20
Sleep disorders	307E, 780F	G47
Attention-deficit/hyperactivity disorder	314	F90
Autism spectrum disorder	299A	F84
Disruptive behavior disorder	-	F91
Personality disorder	301	F60-F62, F69
Substance misuse	291, 303, 304, 305A, 305X	F10-F16, F18, F19
Eating disorder	307B, 307F	F50
Migraine	346A, 346B, 346X, 346W	G43
Epilepsy	345	G40,G41
**Exclusion**		
Congenital Malformation	740A-759X	Q00-Q99

© 2023 Liu S et al. *JAMA Network Open.*

eTable 3. Details on Used Anatomical Therapeutic Chemical (ATC) Codes

Psychotropic Medication	ATC codes
**Antipsychotics**	
**First-generation antipsychotics (FGA)**	
Chlorpromazine	N05AA01
Levomepromazine	N05AA02
Dixyrazine	N05AB01
Fluphenazine	N05AB02
Perphenazine	N05AB03
Prochlorperazine	N05AB04
Thioridazine HCl	N05AC02
Haloperidol	N05AD01
Pipamperon	N05AD05
Flupentixol (2HCL)	N05AF01
Chloroprothixene HCL	N05AF03
Zuclopenthixol	N05AF05
Pimozide	N05AG02
**Second-generation antipsychotics (SGA)**	
Melperon	N05AD03
Sertindole	N05AE03
Ziprasidone	N05AE04
Lurasidone	N05AE05
Clozapine	N05AH02
Olanzapine (Micronized)	N05AH03
Quetiapine (Fumarate)	N05AH04
Sulpiride	N05AL01
Amisulpride	N05AL05
Risperidone	N05AX08
Aripiprazole	N05AX12
Paliperidone	N05AX13
Cariprazine	N05AX15
**Antidepressants**	
**Selective serotonin reuptake inhibitors (SSRI)**	N06AB
**Other antidepressants**	
Non-selective monoamine reuptake inhibitors	N06AA
Monoamine oxidase inhibitors, non-selective	N06AF
Monoamine oxidase A inhibitors	N06AG
Other antidepressants	N06AX
**Anxiolytics**	
Benzodiazepine derivatives	N05BA

© 2023 Liu S et al. *JAMA Network Open.*

Other anxiolytics	N05B, excluding N05BA
**Hypnotics**	
Benzodiazepine derivatives	N05CD
Z-drugs	N05CF
Melatonin	N05CH01
Other hypnotics	N05C, excluding N05CD, N05CF, N05CH01
**Mood stabilizer**	
Carbamazepine	N03AF01
Oxcarbazepine	N03AF02
Valproate	N03AG01
Lamotrigine	N03AX09
Topiramate	N03AX11
Gabapentin	N03AX12
Pregabalin	N03AX16
Lithium	N05AN01
**ADHD medications**	
Guanfacine	C02AC02
Amphetamine	N06BA01
Dexamphetamine	N06BA02
Methylphenidate	N06BA04
Atomoxetine	N06BA09
Lisdexamfetamine	N06BA12

© 2023 Liu S et al. *JAMA Network Open.*

eTable 4. Annual Period Prevalence of Any and Specific Group of Psychotropic Medication Dispensed Among Children (Age 0-11) and Adolescents With Type 1 Diabetes With and Without Type 1 Diabetes From 2006 to 2019

2006	2007	2008	2009	2010	2011	2012	2013	2014	2015	2016	2017	2018	2019	P trend[1]
**Any psychotropic medications**														
**Children with type 1 diabetes**														
0.85 (0.65, 1.10)	1.15 (0.91, 1.43)	1.23 (0.98, 1.52)	1.31 (1.06, 1.62)	1.56 (1.27, 1.88)	1.68 (1.38, 2.03)	1.84 (1.52, 2.22)	2.30 (1.92, 2.73)	2.41 (2.00, 2.87)	2.49 (2.06, 2.98)	2.36 (1.92, 2.88)	2.74 (2.22, 3.33)	2.84 (2.27, 3.52)	3.84 (3.11, 4.69)	<0.01
**Children without type 1 diabetes**														
0.66 (0.65, 0.68)	0.73 (0.72, 0.75)	0.78 (0.76, 0.80)	0.85 (0.83, 0.87)	0.96 (0.95, 0.98)	1.06 (1.05, 1.08)	1.14 (1.12, 1.16)	1.35 (1.32, 1.37)	1.55 (1.53, 1.57)	1.60 (1.58, 1.63)	1.72 (1.69, 1.74)	1.84 (1.82, 1.86)	2.01 (1.99, 2.04)	2.29 (2.27, 2.32)	<0.01
**Adolescents with type 1 diabetes**														
2.72 (2.15, 3.39)	2.98 (2.4, 3.65)	3.42 (2.84, 4.08)	4.91 (4.25, 5.64)	5.55 (4.9, 6.26)	6.28 (5.63, 6.98)	6.69 (6.07, 7.37)	7.53 (6.90, 8.20)	8.82 (8.17, 9.50)	9.75 (9.11, 10.43)	10.75 (10.10, 11.43)	11.65 (11.00, 12.33)	12.78 (12.12, 13.47)	13.54 (12.88, 14.23)	<0.01
**Adolescents without type 1 diabetes**														
1.77 (1.74, 1.79)	2.04 (2.01, 2.07)	2.62 (2.59, 2.65)	3.19 (3.15, 3.22)	3.81 (3.77, 3.84)	4.5 (4.47, 4.54)	5.13 (5.10, 5.17)	5.82 (5.78, 5.85)	6.58 (6.55, 6.62)	7.39 (7.35, 7.43)	8.17 (8.13, 8.21)	8.95 (8.91, 8.99)	9.61 (9.57, 9.65)	10.29 (10.28, 10.36)	<0.01
**First-generation antipsychotics (FGA)**														
**Children with type 1 diabetes**														
0.00 (0.00, 0.05)	0.00 (0.00, 0.05)	0.00 (0.00, 0.05)	0.00 (0.00, 0.05)	0.02 (0.00, 0.08)	0.00 (0.00, 0.06)	0.00 (0.00, 0.06)	0.00 (0.00, 0.07)	0.00 (0.00, 0.07)	0.00 (0.00, 0.08)	0.00 (0.00, 0.09)	0.00 (0.00, 0.11)	0.00 (0.00, 0.13)	0.00 (0.00, 0.15)	-
**Children without type 1 diabetes**														
-	-	-	-	-	-	-	-	-	-	-	-	-	-	-
**Adolescents with type 1 diabetes**														
0.04 (0.00, 0.20)	0.03 (0.00, 0.19)	0.03 (0.00, 0.16)	0.08 (0.02, 0.22)	0.02 (0.00, 0.12)	0.06 (0.01, 0.17)	0.12 (0.05, 0.25)	0.11 (0.04, 0.22)	0.06 (0.02, 0.14)	0.13 (0.06, 0.23)	0.07 (0.03, 0.15)	0.12 (0.06, 0.22)	0.14 (0.07, 0.23)	0.15 (0.08, 0.25)	<0.01
**Adolescents without type 1 diabetes**														

© 2023 Liu S et al. *JAMA Network Open.*

0.03 (0.03, 0.04)	0.02 (0.02, 0.03)	0.03 (0.03, 0.04)	0.04 (0.04, 0.05)	0.05 (0.05, 0.06)	0.07 (0.06, 0.07)	0.07 (0.07, 0.08)	0.08 (0.08, 0.09)	0.09 (0.09, 0.10)	0.10 (0.10, 0.11)	0.10 (0.10, 0.11)	0.12 (0.12, 0.13)	0.12 (0.12, 0.13)	0.13 (0.12, 0.13)	<0.01
**Second-generation antipsychotics (SGA)**														
**Children with type 1 diabetes**														
0.00 (0.00, 0.05)	0.04 (0.01, 0.13)	0.04 (0.01, 0.13)	0.03 (0.00, 0.11)	0.03 (0.00, 0.11)	0.02 (0.00, 0.09)	0.05 (0.01, 0.15)	0.11 (0.04, 0.24)	0.12 (0.04, 0.26)	0.15 (0.06, 0.31)	0.12 (0.04, 0.29)	0.09 (0.02, 0.25)	0.10 (0.02, 0.30)	0.17 (0.05, 0.43)	0.01
**Children without type 1 diabetes**														
0.02 (0.02, 0.02)	0.02 (0.02, 0.03)	0.03 (0.03, 0.03)	0.03 (0.03, 0.03)	0.04 (0.03, 0.04)	0.04 (0.04, 0.04)	0.04 (0.04, 0.05)	0.04 (0.04, 0.05)	0.05 (0.05, 0.06)	0.06 (0.05, 0.06)	0.06 (0.05, 0.06)	0.06 (0.06, 0.06)	0.07 (0.07, 0.08)	0.09 (0.08, 0.09)	<0.01
**Adolescents with type 1 diabetes**														
0.07 (0.01, 0.26)	0.07 (0.01, 0.24)	0.23 (0.10, 0.45)	0.26 (0.12, 0.47)	0.20 (0.09, 0.38)	0.21 (0.11, 0.38)	0.31 (0.18, 0.49)	0.37 (0.24, 0.55)	0.60 (0.43, 0.81)	0.43 (0.30, 0.61)	0.67 (0.51, 0.87)	0.68 (0.52, 0.87)	0.76 (0.60, 0.96)	0.75 (0.59, 0.94)	<0.01
**Adolescents without type 1 diabetes**														
0.14 (0.13, 0.15)	0.16 (0.15, 0.17)	0.20 (0.19, 0.21)	0.24 (0.23, 0.25)	0.30 (0.29, 0.31)	0.35 (0.34, 0.36)	0.41 (0.40, 0.42)	0.49 (0.48, 0.50)	0.58 (0.57, 0.59)	0.65 (0.64, 0.66)	0.71 (0.70, 0.72)	0.78 (0.77, 0.79)	0.86 (0.84, 0.87)	0.93 (0.91, 0.94)	<0.01
**Selective serotonin reuptake inhibitors (SSRI)**														
**Children with type 1 diabetes**														
0.01 (0.00, 0.08)	0.07 (0.02, 0.17)	0.09 (0.03, 0.19)	0.04 (0.01, 0.13)	0.09 (0.03, 0.20)	0.09 (0.03, 0.20)	0.17 (0.08, 0.31)	0.16 (0.07, 0.31)	0.08 (0.02, 0.20)	0.15 (0.06, 0.31)	0.17 (0.07, 0.36)	0.2 (0.08, 0.41)	0.21 (0.08, 0.45)	0.33 (0.14, 0.66)	<0.01
**Children without type 1 diabetes**														
0.02 (0.02, 0.03)	0.02 (0.02, 0.03)	0.02 (0.02, 0.03)	0.03 (0.02, 0.03)	0.03 (0.03, 0.04)	0.04 (0.03, 0.04)	0.05 (0.04, 0.05)	0.05 (0.05, 0.05)	0.06 (0.05, 0.06)	0.06 (0.06, 0.07)	0.07 (0.07, 0.08)	0.08 (0.08, 0.09)	0.10 (0.09, 0.10)	0.12 (0.11, 0.13)	<0.01
**Adolescents with type 1 diabetes**														
0.92 (0.60, 1.30)	0.80 (0.52, 1.19)	1.01 (0.70, 1.40)	1.61 (1.24, 2.06)	1.78 (1.42, 2.22)	2.28 (1.89, 2.73)	2.18 (1.82, 2.59)	2.82 (2.43, 3.25)	3.66 (3.24, 4.12)	3.75 (3.34, 4.19)	4.22 (3.8, 4.67)	4.75 (4.32, 5.21)	5.34 (4.89, 5.80)	5.81 (5.36, 6.28)	<0.01

© 2023 Liu S et al. *JAMA Network Open.*

**Adolescents without type 1 diabetes**														
0.56 (0.55, 0.58)	0.64 (0.63, 0.66)	0.88 (0.86, 0.90)	1.11 (1.09, 1.13)	1.37 (1.35, 1.39)	1.68 (1.65, 1.70)	1.97 (1.95, 1.99)	2.31 (2.29, 2.34)	2.66 (2.63, 2.68)	3.05 (3.03, 3.08)	3.44 (3.41, 3.46)	3.78 (3.76, 3.81)	4.13 (4.10, 4.15)	4.45 (4.42, 4.48)	<0.01
**Other antidepressants**														
**Children with type 1 diabetes**														
0.01 (0.00, 0.08)	0.03 (0.00, 0.10)	0.01 (0.00, 0.08)	0.01 (0.00, 0.08)	0.00 (0.00, 0.06)	0.02 (0.00, 0.09)	0.00 (0.00, 0.06)	0.02 (0.00, 0.10)	0.02 (0.00, 0.11)	0.02 (0.00, 0.12)	0.00 (0.00, 0.09)	0.00 (0.00, 0.11)	0.00 (0.00, 0.13)	0.00 (0.00, 0.15)	0.38
**Children without type 1 diabetes**														
0.01 (0.00, 0.01)	-	-	-	-	0.01 (0.00, 0.01)	0.00 (0,00, 0.01)	-	-	0.01 (0.00, 0.01)	0.00 (0,00, 0.01)	0.01 (0.00, 0.01)	0.01 (0.01, 0.01)	0.01 (0.01, 0.01)	0.90
**Adolescents with type 1 diabetes**														
0.11 (0.02, 0.31)	0.20 (0.07, 0.44)	0.17 (0.06, 0.37)	0.23 (0.11, 0.44)	0.51 (0.33, 0.77)	0.53 (0.35, 0.77)	0.65 (0.46, 0.89)	0.86 (0.65, 1.11)	0.85 (0.65, 1.09)	1.10 (0.88, 1.36)	1.50 (1.25, 1.79)	1.89 (1.62, 2.19)	2.29 (2.00, 2.61)	2.48 (2.18, 2.80)	<0.01
**Adolescents without type 1 diabetes**														
0.07 (0.06, 0.07)	0.08 (0.07, 0.08)	0.18 (0.17, 0.19)	0.28 (0.27, 0.29)	0.40 (0.39, 0.41)	0.55 (0.54, 0.56)	0.72 (0.71, 0.73)	0.90 (0.89, 0.92)	1.10 (1.08, 1.11)	1.31 (1.29, 1.32)	1.52 (1.50, 1.54)	1.73 (1.71, 1.75)	1.93 (1.91, 1.95)	2.13 (2.11, 2.15)	<0.01
**Anxiolytics**														
**Children with type 1 diabetes**														
0.43 (0.28, 0.61)	0.48 (0.33, 0.67)	0.42 (0.28, 0.61)	0.43 (0.29, 0.61)	0.48 (0.33, 0.68)	0.47 (0.32, 0.67)	0.45 (0.30, 0.66)	0.63 (0.44, 0.88)	0.61 (0.42, 0.87)	0.55 (0.35, 0.81)	0.60 (0.38, 0.89)	0.63 (0.40, 0.96)	0.52 (0.29, 0.86)	0.67 (0.38, 1.08)	0.01
**Children without type 1 diabetes**														
0.31 (0.30, 0.32)	0.32 (0.31, 0.33)	0.31 (0.30, 0.32)	0.31 (0.30, 0.32)	0.32 (0.31, 0.33)	0.33 (0.32, 0.34)	0.32 (0.31, 0.33)	0.46 (0.45, 0.47)	0.60 (0.59, 0.61)	0.57 (0.56, 0.59)	0.58 (0.56, 0.59)	0.57 (0.55, 0.58)	0.48 (0.47, 0.49)	0.44 (0.43, 0.45)	0.02
**Adolescents with type 1 diabetes**														
0.88	0.64	0.75	1.13	1.38	1.31	1.65	1.76	2.26	2.72	2.72	2.50	2.94	3.01	<0.01

© 2023 Liu S et al. *JAMA Network Open.*

(0.57, 1.30)	(0.38, 0.99)	(0.49, 1.09)	(0.82, 1.51)	(1.06, 1.77)	(1.01, 1.66)	(1.34, 2.01)	(1.46, 2.11)	(1.93, 2.63)	(2.37, 3.11)	(2.38, 3.09)	(2.18, 2.84)	(2.61, 3.30)	(2.68, 3.36)	
**Adolescents without type 1 diabetes**														
0.40 (0.39, 0.42)	0.45 (0.44, 0.47)	0.65 (0.64, 0.67)	0.82 (0.81, 0.84)	1.06 (1.04, 1.08)	1.30 (1.28, 1.32)	1.50 (1.48, 1.52)	1.78 (1.76, 1.80)	2.08 (2.06, 2.11)	2.34 (2.32, 2.36)	2.49 (2.46, 2.51)	2.61 (2.59, 2.63)	2.62 (2.60, 2.64)	2.62 (2.60, 2.64)	<0.01
**Hypnotics**														
**Children with type 1 diabetes**														
0.04 (0.01, 0.13)	0.10 (0.04, 0.21)	0.15 (0.07, 0.27)	0.22 (0.12, 0.37)	0.32 (0.20, 0.48)	0.33 (0.20, 0.50)	0.40 (0.26, 0.60)	0.49 (0.32, 0.71)	0.63 (0.43, 0.89)	0.85 (0.61, 1.16)	1.09 (0.80, 1.47)	1.50 (1.12, 1.96)	1.73 (1.29, 2.28)	1.92 (1.41, 2.55)	<0.01
**Children without type 1 diabetes**														
0.05 (0.05, 0.06)	0.06 (0.06, 0.07)	0.08 (0.08, 0.09)	0.11 (0.10, 0.12)	0.15 (0.14, 0.15)	0.19 (0.19, 0.20)	0.25 (0.24, 0.25)	0.28 (0.27, 0.29)	0.36 (0.35, 0.37)	0.47 (0.46, 0.48)	0.65 (0.64, 0.66)	0.83 (0.81, 0.85)	1.07 (1.05, 1.09)	1.34 (1.31, 1.36)	<0.01
**Adolescents with type 1 diabetes**														
0.39 (0.19, 0.69)	0.37 (0.18, 0.66)	0.55 (0.33, 0.85)	1.10 (0.80, 1.48)	1.29 (0.98, 1.67)	1.50 (1.19, 1.87)	1.61 (1.31, 1.97)	1.86 (1.54, 2.21)	2.48 (2.13, 2.87)	2.79 (2.43, 3.18)	3.50 (3.12, 3.92)	4.09 (3.69, 4.52)	4.67 (4.25, 5.11)	5.02 (4.60, 5.46)	<0.01
**Adolescents without type 1 diabetes**														
0.28 (0.27, 0.29)	0.33 (0.32, 0.35)	0.53 (0.52, 0.55)	0.75 (0.73, 0.76)	0.97 (0.95, 0.99)	1.21 (1.19, 1.23)	1.43 (1.41, 1.45)	1.61 (1.59, 1.63)	1.88 (1.86, 1.90)	2.15 (2.13, 2.17)	2.60 (2.58, 2.62)	3.05 (3.03, 3.08)	3.42 (3.39, 3.44)	3.77 (3.75, 3.80)	<0.01
**Mood Stabilizer**														
**Children with type 1 diabetes**														
0.37 (0.24, 0.54)	0.38 (0.25, 0.55)	0.29 (0.18, 0.45)	0.34 (0.22, 0.51)	0.38 (0.24, 0.56)	0.38 (0.24, 0.56)	0.30 (0.18, 0.48)	0.33 (0.19, 0.51)	0.36 (0.21, 0.56)	0.33 (0.18, 0.54)	0.25 (0.12, 0.46)	0.49 (0.29, 0.78)	0.55 (0.32, 0.90)	0.33 (0.14, 0.66)	0.44
**Children without type 1 diabetes**														
0.19 (0.18, 0.19)	0.19 (0.18, 0.20)	0.19 (0.18, 0.20)	0.19 (0.18, 0.20)	0.18 (0.18, 0.19)	0.19 (0.18, 0.19)	0.19 (0.18, 0.19)	0.19 (0.18, 0.20)	0.19 (0.18, 0.19)	0.18 (0.17, 0.18)	0.18 (0.17, 0.18)	0.18 (0.17, 0.19)	0.20 (0.19, 0.20)	0.21 (0.20, 0.21)	0.06
**Adolescents with type 1 diabetes**														

© 2023 Liu S et al. *JAMA Network Open.*

0.64 (0.38, 1.00)	0.70 (0.44, 1.07)	0.78 (0.51, 1.13)	0.84 (0.58, 1.18)	0.60 (0.40, 0.87)	0.80 (0.57, 1.08)	0.82 (0.61, 1.09)	0.86 (0.65, 1.11)	0.89 (0.69, 1.14)	1.04 (0.82, 1.29)	1.06 (0.86, 1.31)	1.01 (0.81, 1.24)	1.04 (0.84, 1.26)	1.20 (0.99, 1.43)	<0.01
**Adolescents without type 1 diabetes**														
0.29 (0.28, 0.30)	0.31 (0.30, 0.32)	0.36 (0.35, 0.37)	0.39 (0.38, 0.40)	0.42 (0.41, 0.43)	0.45 (0.44, 0.46)	0.50 (0.49, 0.51)	0.54 (0.53, 0.56)	0.59 (0.58, 0.60)	0.64 (0.63, 0.65)	0.69 (0.68, 0.70)	0.75 (0.74, 0.76)	0.78 (0.77, 0.79)	0.82 (0.81, 0.83)	<0.01
**ADHD medications**														
**Children with type 1 diabetes**														
0.19 (0.10, 0.33)	0.32 (0.20, 0.48)	0.53 (0.37, 0.73)	0.68 (0.50, 0.91)	0.74 (0.55, 0.98)	0.88 (0.66, 1.14)	1.07 (0.83, 1.37)	1.39 (1.10, 1.74)	1.40 (1.10, 1.77)	1.53 (1.19, 1.93)	1.37 (1.03, 1.78)	1.38 (1.02, 1.83)	1.53 (1.11, 2.04)	2.13 (1.59, 2.79)	<0.01
**Children without type 1 diabetes**														
0.20 (0.19, 0.21)	0.26 (0.25, 0.27)	0.32 (0.31, 0.33)	0.39 (0.38, 0.40)	0.50 (0.48, 0.51)	0.58 (0.57, 0.59)	0.65 (0.64, 0.66)	0.71 (0.70, 0.73)	0.76 (0.74, 0.77)	0.79 (0.78, 0.81)	0.83 (0.81, 0.85)	0.87 (0.85, 0.88)	0.99 (0.98, 1.01)	1.19 (1.17, 1.21)	<0.01
**Adolescents with type 1 diabetes**														
0.53 (0.30, 0.87)	0.87 (0.57, 1.27)	1.18 (0.85, 1.59)	1.69 (1.31, 2.14)	1.92 (1.54, 2.36)	2.38 (1.98, 2.83)	2.63 (2.23, 3.07)	2.73 (2.35, 3.16)	3.23 (2.83, 3.67)	3.38 (2.99, 3.80)	3.67 (3.28, 4.09)	3.76 (3.38, 4.18)	3.91 (3.53, 4.32)	4.07 (3.70, 4.48)	<0.01
**Adolescents without type 1 diabetes**														
0.59 (0.58, 0.61)	0.77 (0.76, 0.79)	0.92 (0.90, 0.94)	1.09 (1.07, 1.11)	1.28 (1.26, 1.30)	1.48 (1.46, 1.50)	1.60 (1.58, 1.62)	1.73 (1.71, 1.75)	1.87 (1.85, 1.89)	2.02 (2.00, 2.04)	2.15 (2.13, 2.17)	2.27 (2.25, 2.29)	2.38 (2.36, 2.40)	2.56 (2.54, 2.58)	<0.01

[1] Mann-Kendall trend test.

© 2023 Liu S et al. *JAMA Network Open.*

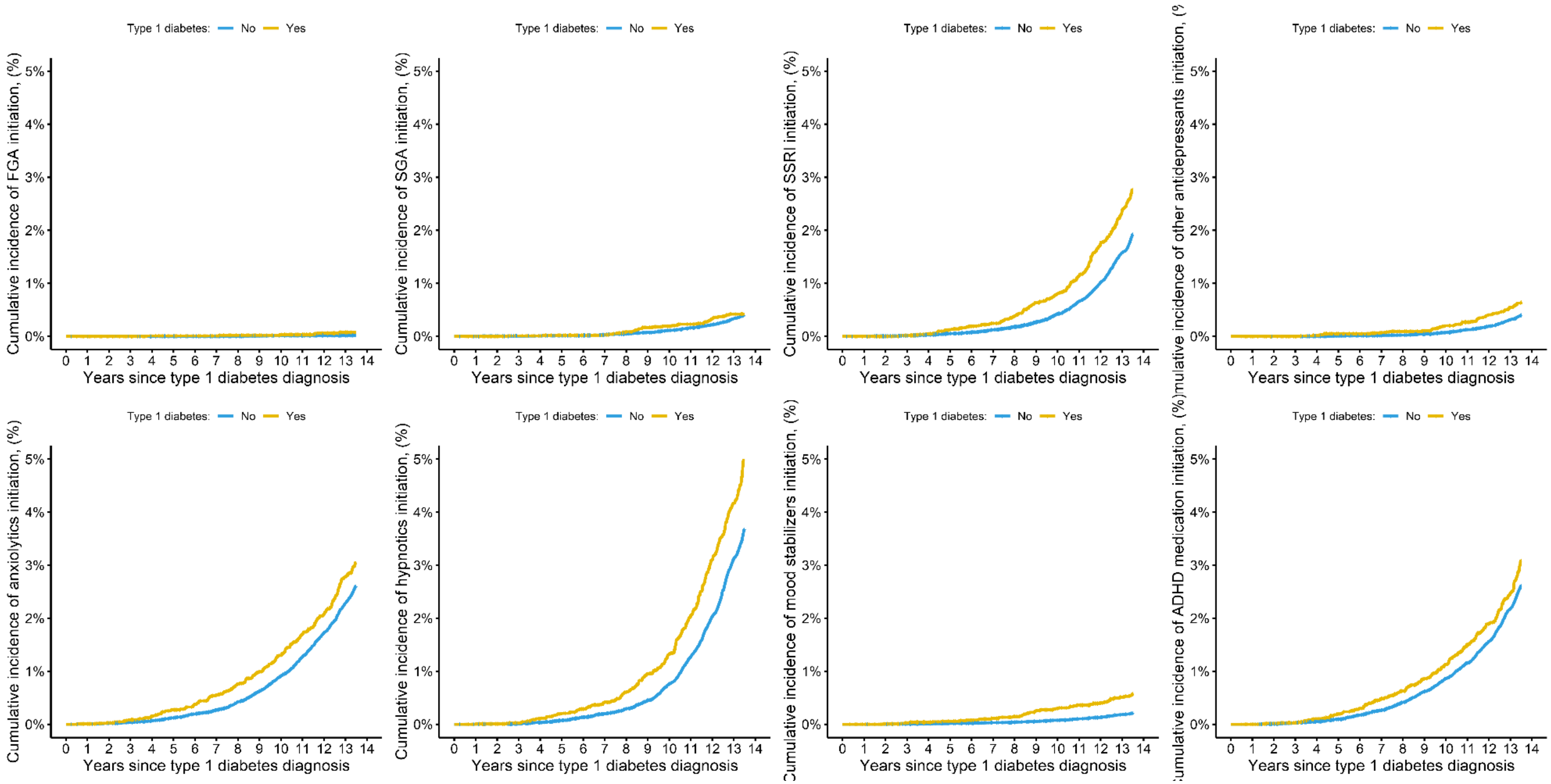

eFigure. Survival Curve of Initiating Specific Psychotropic Medications for Children and Adolescents Diagnosed With Childhood-Onset Type 1 Diabetes Compared With Sex- and Age-Matched Reference Individuals Without Type 1 Diabetes

Initiating medication was defined as a dispensation that occurred by at least 365 days without the dispensation of that medication**.**

© 2023 Liu S et al. *JAMA Network Open.*

eTable 5. Number of Distinct Dispensations of Each Type of Psychotropic Medications in children and Adolescents With Type 1 Diabetes

Psychotropic Medication	ATC codes	N of distinct dispensations by users with T1D
**Antipsychotics**		
**First-generation antipsychotics (FGA)**		
Levomepromazine	N05AA02	30
Dixyrazine	N05AB01	<5
Prochlorperazine	N05AB04	<5
Haloperidol	N05AD01	6
Chloroprothixene HCL	N05AF03	<5
**Second-generation antipsychotics (SGA)**		
Olanzapine (Micronized)	N05AH03	70
Quetiapine (Fumarate)	N05AH04	80
Risperidone	N05AX08	326
Aripiprazole	N05AX12	173
Paliperidone	N05AX13	11
**Antidepressants**		
**Selective serotonin reuptake inhibitors (SSRI)**	N06AB	3368
**Other antidepressants**		
Non-selective monoamine reuptake inhibitors	N06AA	66
Monoamine oxidase A inhibitors	N06AG	<5
Other antidepressants	N06AX	329
**Anxiolytics**		
Benzodiazepine derivatives	N05BA	462
Other anxiolytics	N05B, excluding N05BA	1,249
**Hypnotics**		
Benzodiazepine derivatives	N05CD	159
Z-drugs	N05CF	100
Melatonin	N05CH01	3085
Other hypnotics	N05C, excluding N05CD, N05CF, N05CH01	325

© 2023 Liu S et al. *JAMA Network Open.*

**Mood stabilizer**		
Carbamazepine	N03AF01	96
Oxcarbazepine	N03AF02	160
Valproinsyra	N03AG01	452
Lamotrigine	N03AX09	666
Lithium	N05AN01	23
**ADHD medications**		
Guanfacine	C02AC02	342
Amfetamin	N06BA01	<5
Dexamfetamin	N06BA02	35
Methylphenidate	N06BA04	7,922
Atomoxetin	N06BA09	1,592
Lisdexamfetamin	N06BA12	1,622

© 2023 Liu S et al. *JAMA Network Open.*

eTable 6. Risk of Initiating Psychotropic Medications for children and Adolescents Diagnosed With Type 1 Diabetes Compared to sex- and Age-Matched Reference Individuals Without Type 1 Diabetes [1]

	n (%) of initiators without type 1 diabetes	n (%) of initiators with type 1 diabetes	Hazard Ratio (95%CI) [2]
**N**	98,080	9,808	
**Any psychotropic medication**	4480 (4.3)	525 (5.0)	1.21 (1.11, 1.32)
**Specific psychotropic medication** [3]			
FGA	14 (0.01)	6 (0.1)	-
SGA	293 (0.3)	33 (0.3)	1.13 (0.79, 1.61)
SSRI	1,380 (1.3)	205 (1.9)	1.50 (1.29, 1.73)
Other antidepressants	282 (0.3)	48 (0.5)	1.70 (1.25, 2.31)
Anxiolytics	1,975 (1.9)	239 (2.3)	1.22 (1.07, 1.40)
Hypnotics	2,652 (2.5)	375 (3.6)	1.43 (1.28, 1.59)
Mood stabilizers	162 (0.2)	46 (0.4)	2.85 (2.06, 3.96)
ADHD medications	1,942 (1.8)	232 (2.2)	1.21 (1.06, 1.39)

[1] Initiating medication was defined as a dispensation that occurred by at least 365 days without the dispensation of that medication.
[2] Type 1 diabetes was modelled as a time-varying variable; adjusted for sex, birth cohort, and age at start of follow-up.
[3] FGA, first-generation antipsychotics; SGA, second-generation antipsychotics; SSRI, selective serotonin reuptake inhibitors; ADHD, attention-deficit/hyperactivity disorder.

© 2023 Liu S et al. *JAMA Network Open.*

eReferences

1. Ludvigsson JF, Almqvist C, Bonamy A-KE, et al. Registers of the Swedish total population and their use in medical research. *European journal of epidemiology*. 2016;31(2):125-136.
2. Ludvigsson JF, Andersson E, Ekbom A, et al. External review and validation of the Swedish national inpatient register. *BMC public health*. 2011;11(1):450.
3. Wettermark B, Hammar N, MichaelFored C, et al. The new Swedish Prescribed Drug Register—opportunities for pharmacoepidemiological research and experience from the first six months. *Pharmacoepidemiology and drug safety*. 2007;16(7):726-735.

© 2023 Liu S et al. *JAMA Network Open.*
