# Supplementary material for: Psychotropic Medication Use in Children and Adolescents With Type 1 Diabetes
Source: JAMA Netw Open. 2023 Oct 3;6(10):e2336621. doi: 10.1001/jamanetworkopen.2023.36621 (PMC10548296; doi:10.1001/jamanetworkopen.2023.36621)
Supplement: Supplement 2. — Data Sharing Statement [file jamanetwopen-e2336621-s002.pdf]

Data Sharing Statement

Liu. Psychotropic Medication Use in Children and Adolescents With Type 1 Diabetes. *JAMA Netw Open*. Published October 03, 2023. doi:10.1001/jamanetworkopen.2023.36621

Data

Data available: The data that support the findings of this study are available from the Swedish National Board for Health and Welfare, but restrictions apply to the availability of these data, which were used under license for the current study, and so are not publicly available. Data are, however, available from the authors upon reasonable request and with permission of the Swedish National Board for Health and Welfare.
